# Extended Distal Pancreatectomy with En Bloc Resection of the Celiac Axis for Locally Advanced Pancreatic Cancer: A Case Report and Review of the Literature

**DOI:** 10.1155/2012/543167

**Published:** 2012-04-11

**Authors:** Patrick H. Alizai, Andreas H. Mahnken, Christian D. Klink, Ulf P. Neumann, Karsten Junge

**Affiliations:** ^1^Department of General, Visceral and Transplantat Surgery, University Hospital of the RWTH Aachen, Pauwelsstr 30, 52074 Aachen, Germany; ^2^Department of Diagnostic and Interventional Radiology, University Hospital of the RWTH Aachen, Pauwelsstr 30, 52074 Aachen, Germany

## Abstract

Due to a lack of early symptoms, pancreatic cancers of the body and tail are discovered mostly at advanced stages. These locally advanced cancers often involve the celiac axis or the common hepatic artery and are therefore declared unresectable. The extended distal pancreatectomy with en bloc resection of the celiac artery may offer a chance of complete resection. We present the case of a 48-year-old female with pancreatic body cancer invading the celiac axis. The patient underwent laparoscopy to exclude hepatic and peritoneal metastasis. Subsequently, a selective embolization of the common hepatic artery was performed to enlarge arterial flow to the hepatobiliary system and the stomach via the pancreatoduodenal arcades from the superior mesenteric artery. Fifteen days after embolization, the extended distal pancreatectomy with splenectomy and en bloc resection of the celiac axis was carried out. The postoperative course was uneventful, and complete tumor resection was achieved. This case report and a review of the literature show the feasibility and safety of the extended distal pancreatectomy with en bloc resection of the celiac axis. A preoperative embolization of the celiac axis may avoid ischemia-related complications of the stomach or the liver.

## 1. Introduction

Pancreatic adenocarcinoma is still a tumor entity with a poor prognosis and an overall long-term survival of 1–5% [1, 2]. A major problem is its early detection since 80–90% of the pancreatic cancers are locally or systemically advanced at the time of diagnosis [2–4]. Despite recent advances in chemotherapy, complete resection remains the only chance for cure [3, 5]. Thus, five-year survival rates of about 25% are observed if patients underwent surgery [2]. Especially cancers of the body and tail of the pancreas are discovered mostly at advanced stages due to a lack of early symptoms; for example, obstructive jaundice which is caused by cancers of the head of the pancreas. These locally advanced cancers of the body and tail often involve major vessels such as the celiac axis or the common hepatic artery [6]. Hence the tumors are declared unresectable in spite of the absence of distant metastasis.

The standard resection for tumors of the body and tail of the pancreas is a distal pancreatectomy with concomitant splenectomy [7]. Distal pancreatectomy with en bloc resection of the celiac artery has broadened the operative spectrum in pancreatic surgery. First reported by Appleby in 1953 to achieve complete nodal clearance around the celiac artery for advanced gastric cancer, Nimura adopted this approach, for advanced cancers of the pancreatic body [6, 8]. Since then just a few of these procedures have been described and due to the small number of patients, the overall survival benefit is still unknown [9–11]. We present the case of a 48-year-old female with pancreatic body cancer invading the celiac axis treated by preoperative embolization and subsequent extended distal pancreatectomy and splenectomy with en bloc resection of the celiac axis.

## 2. Case Presentation

**Figure 1:**
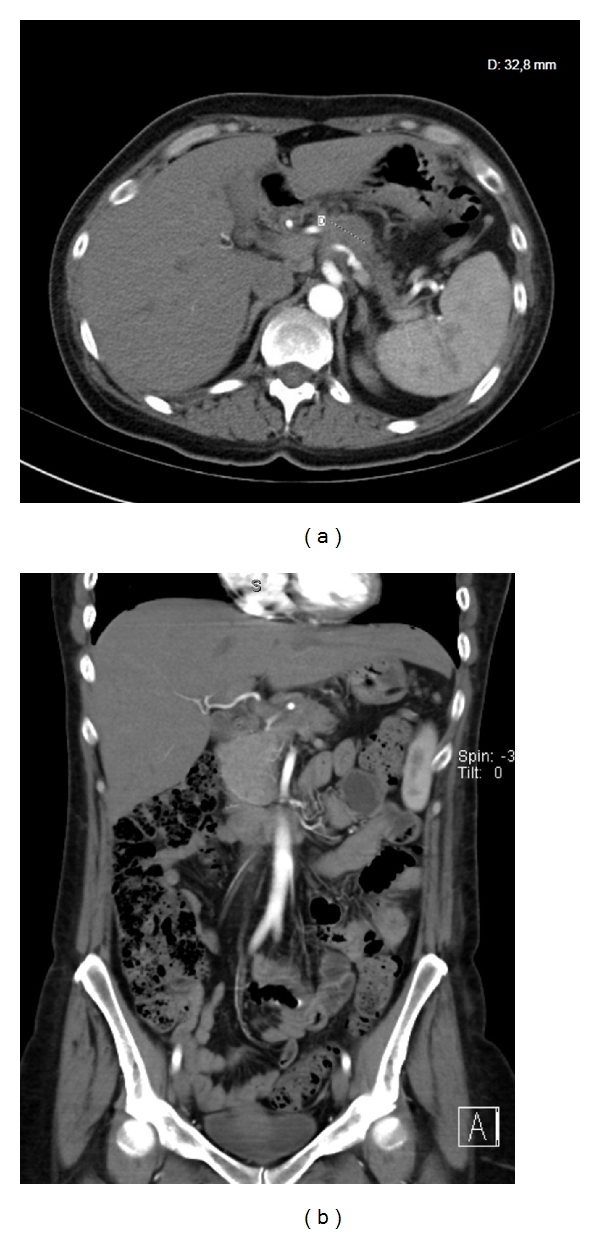
(a) Preoperative arterial phase contrast-enhanced CT scan showing a 3.3 cm lesion in the body of the pancreas. (b) The coronary slice displays tumor infiltration of the celiac trunk, and the superior mesenteric artery is not involved.

**Figure 2:**
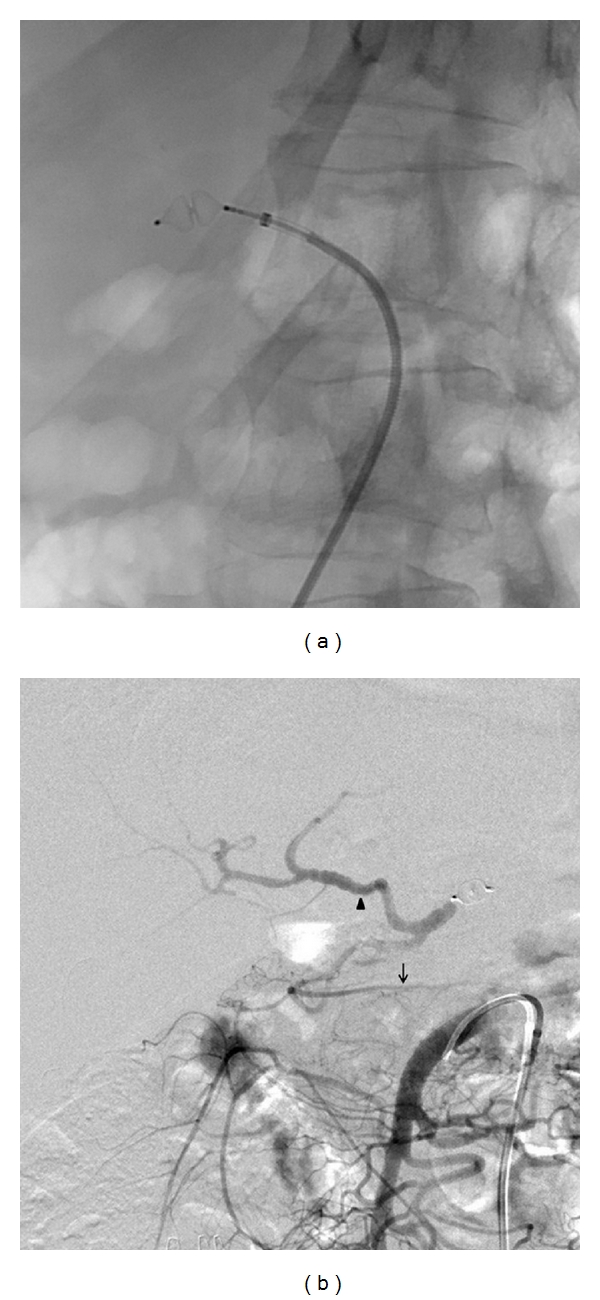
(a) Embolization of the celiac axis with an 8 mm Amplatzer vascular plug 4. (b) The immediate angiographic control after embolization shows a collateral circulation from the superior mesenteric artery via the pancreatoduodenal arcades to the gastroduodenal artery. This ensures sufficient arterial flow to the right gastroepiploic artery (arrow) and the proper hepatic artery (arrowhead).

A 48-year-old otherwise healthy woman had suffered epigastric and back pain for 4 weeks. At first, gastroscopy was performed showing no pathological findings. Subsequent contrast-enhanced computed tomography depicted a 3.3 cm lesion in the body of the pancreas with invasion of the celiac axis (Figure 1(a)). Therefore, the tumor was declared unresectable in an external hospital, and a palliative chemotherapy with gemcitabine and erlotinib was initiated. After 3 cycles of chemotherapy, she presented to our clinic to be reassessed. We carefully reviewed the findings and agreed a surgical approach with her. The patient underwent laparoscopy to exclude hepatic and peritoneal metastasis. Subsequently a selective embolization of the common hepatic was performed. First celiac and superior mesenteric artery angiograms were obtained to confirm the presence of the pancreatoduodenal arcade. Thereafter, a 6F sheath was placed in the common hepatic artery. After the identification of the origin of the gastroduodenal artery an 8 mm Amplatzer vascular plug 4 (AGA Medical Corp., Plymouth, MN USA) was placed in the common hepatic artery (Figure 2(a)). Completion angiograms of the celiac trunk and the superior mesenteric artery confirmed complete occlusion of the common hepatic artery and retrograde arterial perfusion of the liver and the stomach through the pancreatoduodenal arcade (Figure 2(b)).

**Figure 3:**
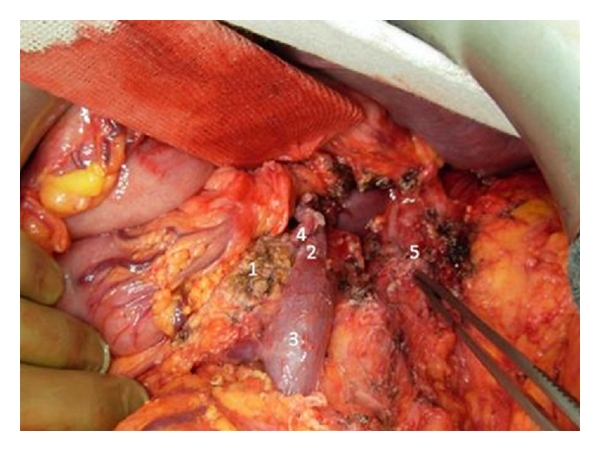
Operative photograph taken after the removal of the specimen. 1: head of the pancreas, 2: portal vein, 3: superior mesenteric vein, 4: gastroduodenal artery, and 5: celiac axis stump.

Fifteen days after embolization of the celiac axis, the extended distal pancreatectomy with splenectomy and en bloc resection of the celiac axis was carried out. We favour an upper abdominal transverse incision with extension to the xiphoid to ensure maximum visibility and access to the pancreas. After confirming the absence of hepatic and peritoneal metastasis, the superior mesenteric artery was exposed at its origin from the aorta to exclude tumor infiltration. The patient underwent a prophylactic cholecystectomy to avoid postoperative ischemic cholecystitis. The hepatoduodenal ligament was dissected, and the proper hepatic artery, the common hepatic artery, and the gastroduodenal artery were exposed. After the common hepatic artery was divided proximally to the origin of the gastroduodenal artery, an excellent pulsation of the left and right hepatic artery was still assessed. The pancreas was transected at the level of the portal vein. The dissection of the portal vein revealed a potential tumor infiltration at the splenic vein confluence; thus, a concomitant portal vein wedge resection was performed. Then the origin of the celiac artery was identified above the superior mesenteric artery, and the celiac artery and the left gastric artery were divided. The body and tail of the pancreas, the spleen, the celiac axis, and the surrounding lymph nodes and nerve plexus were removed en bloc (Figure 3). Finally, the pancreatic duct was ligated separately and the pancreatic head stump was oversewn with nonabsorbable suture material. The operation lasted 195 min, the intraoperative blood loss was 350 mL, and no red blood cell transfusions were required.

**Figure 4:**
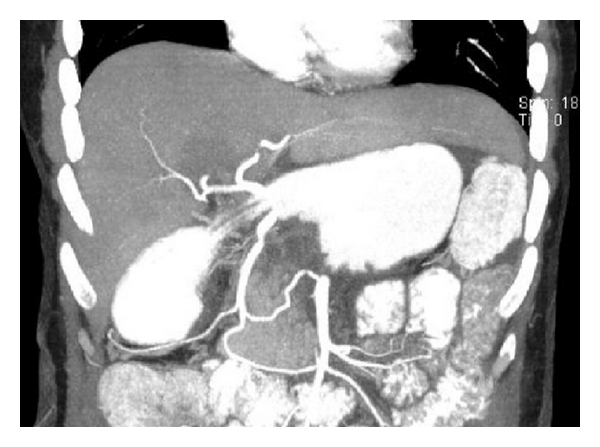
The postoperative arterial contrast-enhanced CT scan shows an excellent arterial flow to the hepatobiliary system via gastroduodenal artery and pancreatoduodenal arcades from the superior mesenteric artery.

The postoperative course went uneventful, and the patient was discharged after 20 days stay in the hospital. A postoperative CT scan revealed sufficient arterial flow of the hepatic arteries via the pancreatoduodenal arcades (Figure 4). A moderate increase of the serum concentrations of alanine aminotransferase and aspartate aminotransferase returned to normal ranges within the hospital stay. A postoperative diarrhoea or ischemic gastropathy did not occur. Additionally, the patient did not develop a pancreoprivic diabetes mellitus. The postsplenectomy vaccine prophylaxis against *Streptococcus pneumoniae*, *Haemophilus influenzae* type B, and *Neisseria meningitidis* was administered during, the hospital stay.

The histopathologic findings showed an invasive ductal adenocarcinoma of the pancreas (pT3) with a size of 6 cm and clear histologically surgical margins (R0). The histopathologic grading was G2 (moderately differentiated), and two regional lymph node metastases were detected (2/17). A notable perineural infiltration extending to the celiac plexus was present. On the recommendation of the interdisciplinary gastrointestinal tumor board, a postoperative chemotherapy with gemcitabine (1000 mg/m^2^ for 3 weeks of a 4-week cycle for 6 months) was carried out. After treatment completion no local recurrence and distant metastasis occurred as evaluated by CT scan.

## 3. Discussion

Due to late presentation of symptoms, more than 75% of carcinomas of the body and tail of the pancreas are considered unresectable at the time of diagnosis [12–14]. The most common reasons are the presence of hepatic metastases, peritoneal dissemination of tumor, or the direct invasion of major vascular structures, respectively. Venous resections including the portal and superior mesenteric vein are well-accepted procedures, while tumor invasion of the superior mesenteric artery or the celiac axis is regarded as a contraindication for a surgical approach [1, 2]. This rejection is due to previously reported high mortality and poor prognosis after resection of arterial vessels [3, 15]. Should a tumor invasion of the common hepatic artery or the celiac axis be indeed a general contraindication to resection? Consequentially, these patients, as seen in our case report, would receive chemotherapy in a palliative intention with an approximately median survival of only 6 months [16].

Since Nimura implemented the distal pancreatectomy with en bloc resection of the celiac axis in 1976, a few authors have performed this procedure successfully [9–11]. Due to the small number of patients, the overall survival benefit remains unclear. Hirano et al. report an estimated overall 1- and 5-year survival rate of 71% and 42%, respectively, and the median survival was 21.0 months [10]. Mayumi et al. compared the outcome of 47 patients with carcinoma of the pancreatic body or tail [9]. Six cases underwent an extended distal pancreatectomy with en bloc resection of the celiac axis, 19 cases received a standard distal pancreatectomy, and a third group consisted of 22 patients with unresectable pancreatic adenocarcinoma. The cumulative 1- and 3-year accumulated survival rates for the extended, standard, and unresectable groups were 40.0, 33.3, and 5.4, and 20.0, 16.6, and 0%, respectively. Wu et al. compared 31 patients with invasion of the common hepatic artery or celiac axis: 11 patients, who received a distal pancreatectomy with en bloc resection of the celiac axis, to 20 patients, who did not undergo surgery [11]. The first group had a significantly prolonged median survival time compared with the second group (14 versus 5 months, *P* = 0.013). However, estimated 5-year survival rates of 42% as reported by Hirano et al. [10] must be read with caution due to the small number of patients. But a median survival time of 21.0 months is excellent in cases that have previously been considered unresectable. The distal pancreatectomy with en bloc resection of the celiac axis can achieve a R0 resectability rate of 91% [10]. It is well known that a complete tumor resection with negative surgical margins is an important independent prognostic factor for survival [13] and the only chance for cure [3, 5]. Hence, a surgical approach is justified in selected cases with advanced pancreatic cancers.

Additionally, this operation not only increases the resectability but also improves quality of life due to long-lasting pain relief [11, 17]. Advanced pancreatic adenocarcinoma frequently involves nervous tissue and spreads along nerve fibers [18, 19]. The cancer involvement of the celiac plexus and celiac ganglions results in intractable pain, and medical treatment is often unsuccessful [17]. Thus new techniques including celiac plexus block [17, 20, 21], intrathecal morphine administration via a subcutaneous pump [22], or thoracoscopic splanchnicectomy [23] have been developed. However complete pain relief is difficult to achieve. Hirano et al. report a complete pain relief in all 10 patients with preoperative intractable pain after distal pancreatectomy with en bloc resection of the celiac axis [10]. Because radical distal pancreatectomy with en bloc resection of the celiac axis includes complete removal of the celiac plexus and celiac ganglions, it is understandable that the operative procedure results in excellent pain control [6, 17, 24].

Despite these advantages the high morbidity rate of the distal pancreatectomy with en bloc resection of the celiac axis must be considered. Hirano et al. report a morbidity rate of 48% in a series with 23 patients [10]. The main postoperative complications were pancreatic fistula and ischemic gastropathy [10, 25, 26]. An arterial reconstruction is mostly dispensable because collateral pathways via the superior mesenteric artery, pancreatoduodenal arcades, and gastroduodenal artery maintain the arterial blood supply for the hepatobiliary system [6]. The entire stomach can be preserved because collateral pathways ensure arterial flow also to the right gastroepiploic artery [6]. A preoperative embolization of the celiac axis may prevent ischemia-related complications of the stomach or the liver by enlarging these collateral pathways from the superior mesenteric artery. Hirano et al. noticed a remarkable decrease of ischemic gastropathy to an incidence of 13% after the introduction of the preoperative coil embolization [10]. Therefore, we also performed an embolization of the celiac axis 15 days prior to the operation. Previously published case series report a waiting period between embolization and surgery of 1–29 days [10, 27, 28]. Although there is no reliable evidence base available regarding length of waiting period after embolization, we have some knowledge from studies concerning preoperative gastric conditioning prior to esophagectomy. Bludau et al. found that gastric mucosal oxygen saturation, which drops significantly with devascularization of the stomach, nearly returns to baseline levels by 4-5 days [29]. A study by Veeramootoo et al. on gastric conduit reported that ischemic preconditioning 2 weeks before surgery was associated with less ischemia-related gastric failure compared to 5 days prior to surgery [30]. However, the ideal timing needs to be further ascertained.

During surgery the key vessel of the distal pancreatectomy with en bloc resection of the celiac axis is the inferior pancreatoduodenal artery from the superior mesenteric artery, and surgeons should identify this artery to avoid an accidental injury. If an arterial reconstruction is needed, a middle colic artery-gastroepiploic artery bypass can be used [10]. The splenic artery, which can be taken from the resected specimen, is another suitable option for reconstruction [26]. We performed a staging laparoscopy before coil embolization of the celiac axis to confirm absence of metastatic disease. Approximately 40–50% of the patients have distant metastases at the time presentation [2, 31, 32]. Despite advances of imaging technology, noninvasive staging modalities are still inaccurate in identifying small volume metastatic disease [32]. A staging laparoscopy may reveal these unsuspected metastases and prevent unnecessary coil embolization and laparotomy.

This case report and the cited studies display the feasibility and safety of the operation. The encouraging results of Hirano et al. [10], who reported a median survival time of 21 months, cannot hide the fact that distal pancreatectomy with en bloc resection of the celiac axis on its own cannot achieve a favorable long-term survival. Despite the excellent local control achieved through this procedure, early hepatic recurrence still persists [10]. The adjuvant treatment as a postoperative chemotherapy seems to be a key factor to further improve patient survival [2].

## 4. Conclusion

The extended distal pancreatectomy with en bloc resection of the celiac axis following a preoperative embolization provides a high resectability rate and a long-lasting pain relief. These results justify further evaluation of the long-term survival benefits. However, this procedure should always be considered in patients with locally advanced cancer of the pancreatic body or tail involving the celiac axis or the common hepatic artery. The presentation of these patients to a high volume center of pancreatic surgery may avoid premature palliative chemotherapy.
